# Moderate irrigation intervals facilitate establishment of two desert shrubs in the Taklimakan Desert Highway Shelterbelt in China

**DOI:** 10.1371/journal.pone.0180875

**Published:** 2017-07-18

**Authors:** Congjuan Li, Xiang Shi, Osama Abdalla Mohamad, Jie Gao, Xinwen Xu, Yijun Xie

**Affiliations:** 1 Department of National Engineering Technology Research Center for Desert-Oasis Ecological Construction, Xinjiang Institute of Ecology and Geography, Chinese Academy of Sciences, Urumqi, Xinjiang, China; 2 Department of Agriculture College, Shihezi University, Shihezi, Xinjiang, China; 3 Department of Key Laboratory of Biogeography and Bioresource in Arid Land, Xinjiang Institute of Ecology and Geography, Chinese Academy of Sciences, Urumqi, Xinjiang, China; 4 Department of Midong Municipal Bureau of Parks, Urumqi Forestry Bureau, Urumqi, Xinjiang, China; University of Vigo, SPAIN

## Abstract

**Background:**

Water influences various physiological and ecological processes of plants in different ecosystems, especially in desert ecosystems. The purpose of this study is to investigate the response of physiological and morphological acclimation of two shrubs *Haloxylon ammodendron* and *Calligonum mongolicunl* to variations in irrigation intervals.

**Methodology/Principal findings:**

The irrigation frequency was set as 1-, 2-, 4-, 8- and 12-week intervals respectively from March to October during 2012–2014 to investigate the response of physiological and morphological acclimation of two desert shrubs *Haloxylon ammodendron* and *Calligonum mongolicunl* to variations in the irrigation system. The irrigation interval significantly affected the individual-scale carbon acquisition and biomass allocation pattern of both species. Under good water conditions (1- and 2-week intervals), carbon assimilation was significantly higher than other treatments; while, under water shortage conditions (8- and 12-week intervals), there was much defoliation; and under moderate irrigation intervals (4 weeks), the assimilative organs grew gently with almost no defoliation occurring.

**Conclusion/Significance:**

Both studied species maintained similar ecophysiologically adaptive strategies, while *C*. *mongolicunl* was more sensitive to drought stress because of its shallow root system and preferential belowground allocation of resources. A moderate irrigation interval of 4 weeks was a suitable pattern for both plants since it not only saved water but also met the water demands of the plants.

## Introduction

As one of the main determinants of the types and distribution of global vegetation, water influences various physiological and ecological processes of plants in different ecosystems [1–4]. The water scarcity is becoming a world-wide problem of increasing severity due to insufficient precipitation in desert ecosystems or extreme arid region [4–5]. To overcome such shortage, lower-quality water of saline-alkaline groundwater is widely used [6–9], which is running the risk of soil salinization and greater salinity hazards to plant growth and survival in irrigated areas [10–11]. However, drought is the crucial limiting factor for plant recruitment, carbon fixation, growth, nutrient dynamics and survival in these region [3, 5, 12–13]. In other words, understanding the plant–water relationship at the morphological and physiological scale is urgent for establishing a suitable irrigation program for artificial vegetation growth and survival in extremely arid areas.

**Fig 1:**
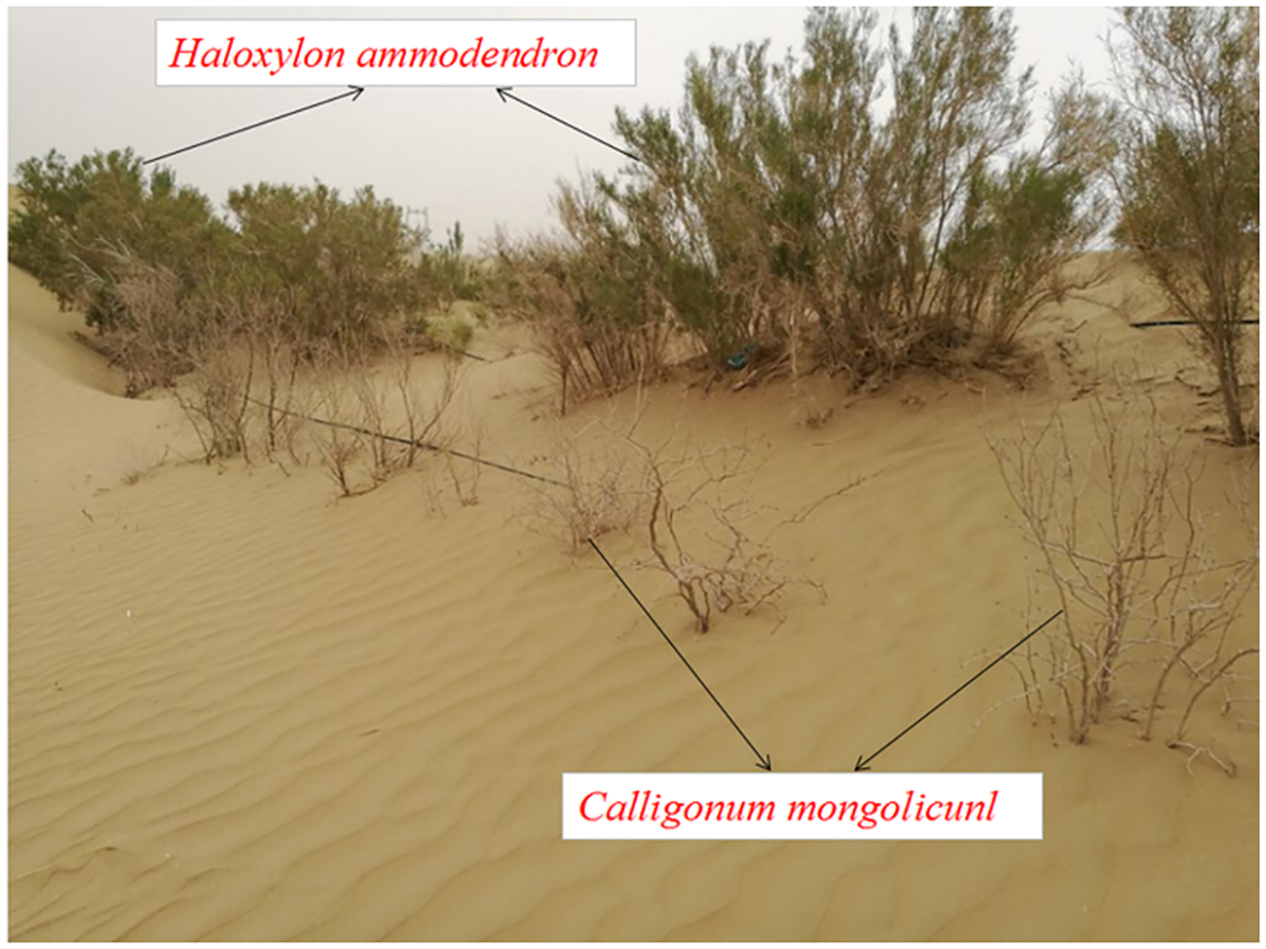
Growth status of *Haloxylon ammodendron* and *Calligonum mongolicunl* in parts of the Taklimakan Desert Highway Shelterbelt.

The Taklimakan Desert is the largest mobile desert in China and located in the hinterland of the Tarim Basin, where the precipitation is less than 50 mm per year, and no snow cover in winter, while annual pan evaporation is more than 3000 mm [5]. It is extremely arid that has been called the “Dead Sea” since few organism exist in this harsh environment [14]. The Taklimakan Desert Highway Shelterbelt (TDHS) and Taklimakan Desert Botanic Garden (TDBG) were constructed in 2003 to limit sand drift along the Taklimakan Desert Highway. After 6 years’ (from 1997 to 2002) introduction and selection, several droughts- and salt-tolerant plants of three families of *Haloxylon Bunge*, *Calligonum Linn*, and *Tamarix Linn* have been introduced to this area, while the *Tamarix Linn* was just a small quantity. *Haloxylon ammodendron* and *Calligonum mongolicunl* were two main species along the TDHS, and saline groundwater with drip-irrigation was used for artificial shelterbelt [5], which typically contains solutes of varying concentrations but widely used in drought environments [15–17]. Along the whole TDHS, the *H*. *ammodendron* grows well, while *C*. *mongolicunl* in some parts of TDHS has been dead due to irregular or insufficient irrigation (Fig 1). Such phenomena may reflect the different adaptability or adaptive strategies of the two species. As a result, understanding the adaptive strategy of the two species under irrigation in extreme environment will be useful to determine suitable amounts of irrigation for this area, and ultimately, to ensure plant survival and prevent soil salinization and plant salt toxicity.

Desert plants adapted to drought stress for extended periods of time usually possess high convergent adaptation such as the similar morphology, internal structures, and physiological characteristics [1, 4, 18]. However, the phenotypic character is the result of the interaction of genetic and environmental factors, in this case, the adaptive process may be diverse since the different genetic basis of each species [19–22]. Particularly, desert plants are introduced into the extreme environments, and the adaptation and responses of plants to environmental stresses are variable [4]. Li et al. (2005) showed that xerophytes in natural desert possess different root morphology and water use strategies, while the physiological characteristics such as leaf photosynthetic intensity show a remarkable consistency to water conditions [21].

*H*. *ammodendron* and *C*. *mongolicunl* are the dominant xerophyte species of a large ecological importance and are crucially important in stabilizing of sand dunes. The two species share some similarities: both of them are shrubs and exhibit a much higher tolerance to water shortage than other plants [18], and both have photosynthetic pathway of C_4_ [23, 24]; both species have large root systems, and *H*. *ammodendron* could penetrate to 10 m below the soil surface and reach the groundwater [24–25], while for *C*. *mongolicunl*, the roots were much larger, which mainly extend horizontally about 3 m in vertical direction and about 10–20 m in horizontal direction [5, 26–27]. Additionally, the two species distribute differently in their natural habitats. *H*. *ammodendron* mainly grows on inter-dune lowland and the flat slope of dunes where soil water and nutrient are relatively higher than the top dune [26, 28], while *C*. *mongolicunl* mainly grows on the top of sand dune or semi-dune, and they rarely grow together in natural environment in Gurbantunggut Desert [29, 30]. Compared with *H*. *ammodendron*, *C*.*mongolicunl* is more tolerant to drought but less tolerant to salinity in the natural habitat [31, 32].

Except for the preferential allocation of photoassimilates to the belowground root system in response to drought [28, 33–35], defoliation is also a strategy to drought tolerance for desert plants, which results in insufficient photosynthesis and leads to carbon starvation [26]. Conversely, leaf enlargement is another way to gain more carbon assimilation products and increase water-use efficiency (WUE) for plants [36]. Additionally, their metabolism can still be vulnerable to variation in soil water availability [37–38]. The main physiological parameters, such as photosynthetic rate (*P*_*n*_) and transpiration rate (*T*_*r*_), are variably inhibited during times of water shortage. In this case, comparing the morphological and physiological characteristics together to determine the water use patterns of the two species is urgent for the survival and sustainable development of the artificial shelterbelt.

Given the known differences in their natural habitats and root morphology [23, 39], the differences in their growth and survival conditions along the TDHS, we assume that the morphological adaptation to the absence of water or adverse climate conditions may be the major cause of the death of *C*. *mongolicunl* and *C*. *mongolicunl* is more sensitive to irrigation water. Moreover, we hypothesize that moderate irrigation intervals facilitate the establishment of two species in the Taklimakan Desert Highway Shelterbelt since it not only saved water but also met the water demands of the plants.

## Materials and methods

### Study area

Since the crude management and the rough experimental conditions, it is impossible to carry out the strict and fine research along the TDHS, only some investigations for plants growth, survival, soil and water can be carried out, and some strict and fine controlled research was carried out in the TDBG. Therefore, the study was carried out in the TDBG (39°00′N, 83°40′E), and the TDHS (37°08′N, 81°24′E) in China respectively, without any ethical or legal restrictions. Thus all data can be fully available and shared for any other study or those readers who are interested in this study. And the study areas were not the national park or other protected area of land or sea, and no specific permissions were required for these locations.

Taklimakan Desert Botanic Garden (TDBG), which is also named Tazhong Station of Desert Research, authorized and founded by the Chinese government, and the main task is to carry out ecological researches of Taklimakan Desert Highway Shelterbelt, the study areas were not the national park or other protected area of land or sea, and no specific permissions were required for these locations.

The Taklimakan Desert is the extremely arid region in China, which has been called the “Dead Sea” since no organism lives under its harsh environment characterized by very limited rainfall and strong evaporative potential [6,14]. Unlike Gurbantunggut Desert, the native habitat of *H*. *ammodendron* and *C*. *mongolicunl*, where there is a few effective rainfall of 150–200 mm per year, and annual pan evaporation is around 1000 mm. In addition, there is stable snow layer of 30–50 cm in thick in winter. To ensure the survival of artificial shelterbelts at the Taklimakan Desert Highway Shelterbelt (TDHS) and Taklimakan Desert Botanic Garden (TDBG), low-quality water of saline groundwater, which typically contains solutes of 2.8–29.7 g L^−1^ salt concentrations used along the TDHS [5, 40]. While in some parts of the TDHS, *C*. *mongolicunl* has been dead, *H*. *ammodendron* grows well, and the growth states of two species in some areas along the TDHS can be seen in (Fig 1).

The study was carried out in the Taklimakan Desert Botanic Garden (TDBG) (39°00′N, 83°40′E), and the Taklimakan Desert Highway Shelterbelt (TDHS) (37°08′N, 81°24′E) in China respectively, without any ethical or legal restrictions. Thus, the data can be available and shared for any other study or readers who are interested in this study. Both of the study areas are located in the hinterland of Taklimakan Desert [14, 40]. The TDBG and TDHS were completed in 2003, and *H*. *ammodendron* and *C*. *mongolicunl* are two xerophytes that grow well in this area [40]. The row and line spacing for the plants were 1 and 3 m respectively. Saline groundwater (4 g L^−1^) was used for drip-irrigation extracted from the saline groundwater well in TDBG and TDHS respectively, and the initial irrigation system was applied at 2-week intervals from March to October during vegetation periods every year, and December to February of the next year were the non-irrigation time since such period was not the water shortage period with small evaporation and lower temperature [5, 9,14]. The soil is typically sandy with a limited nutrient content, and without any fertilizing materials applied to the ground soil. The soil fundamental physical properties of shifting sand and shelterbelt soil after the plants had been set up for 7 years (2012) which showed that the shelterbelt soil properties were significantly improved (Table 1).

**Table 1:** Soil physical properties of shifting sand and shelterbelt soil after shelterbelt has been set up for 7 years.

Physical properties	Soil Bulk Density (g cm3-)	Clay Content (%)	Silt Content (%)	Sand Content (%)	Soil aggregate sizes (%)					Soil aggregate stability		
					>5 mm	5~2 mm	2~1 mm	1~0.5 mm	0.5~0.25 mm	>0.25 mm	MWD (mm)	GMD (mm)
Shifting sand (CK)	1.5±0.03a	0.27±0.01	12.35±0.04	87.48±1.25	0b	0b	0b	0b	0b	0b	0.13b	0.13b
Shelterbelt soil	1.3±0.007b	0.67±0.01a	19.9±0.37a	79.69±7.25b	4.08±0.09a	1.25±0.02a	0.33±0.01a	0.25±0.01a	0.18±0.01a	6.08±0.11a	0.48±0.01a	0.16±0.001a

Note: MWD presents the mean weight diameter, GMD presents geometric mean diameter and CK presents the shifting sand, the different letters give significant difference at *P* = 0.05.

### Experiment design

Since *C*. *mongolicunl* has been dead in some parts of the TDHS under 2 weeks interval, 1 week and 2, 4, 8 and 12 weeks range of five treatments drip-irrigation frequencies from March to October during 2012–2014 were conducted for *H*. *ammodendron* and *C*. *mongolicunl* at the TDBG, the saline groundwater (4 g L^−1^) was extracted from the groundwater well at the TDBG, with the same amount of water (about 1250 m^3^ hm^-2^) used every time in 12 h of continuous irrigation [41].

### Root and shoot distribution investigation

Since there is no significant difference for root depth, shoots, and roots biomass allocation were presented between different treatment of 1, 2, 4, 8 and 12W at August 2014, the further investigation of biomass allocation have been conducted in March 2016 (2 W) again both at TDBG and the area where some *C*. *mongolicunl* have been dead along the TDHS respectively by excavation and division of whole plants as previously reported by Xu and Li 2006 [35], with the entire root system divided into feeder roots and primary roots, and the shoot divided into assimilating branch and other parts. For each species, five repetition plants of average size in each treatment or study area were randomly selected for root and shoot biomass evaluation, with average height and canopy size of the *H*. *ammodendron* and *C*. *mongolicunl* being 1–2 m height and 1–2 m × 1–2 m in the canopy, respectively. The roots were washed, and all the parts of the plant were oven-dried and used for evaluating the biomass allocation.

### Soil sampling

The detailed procedure of soil sampling measurements were performed as previously described [35] At the same time, soil sampling of five repetitions was conducted in August 2014 after the experiment had been carried out for 2 years at both before and after irrigation in each treatment for soil water content analysis. The large ground soil cylinder surrounding the primary root was removed manually at 5-cm intervals for 0–10 cm soil depth since there were salt crusts in the soil surface, and 10-cm intervals for 10–200 cm.

### Measurement of photosynthesis (*P_n_*), transpiration rate (*T_r_*) and leaf water potential (*Ψ_l_*)

During the whole growing season of the studied species in 2014 from the May 6^th^ to August 13^th^ (127–226 Julian d), we selected the youngest mature and healthy assimilative organs for measuring *P*_*n*_, *T*_*r*_ and leaf water potential (*Ψ*_*l*_). We measured the diurnal course of *P*_*n*_ and *T*_*r*_ of the two species using a Li-6400 portable photosynthesis system (LICOR, Lincoln, NE, USA) at the day before and after irrigation in each treatment on sunny days. The detailed measurement procedure was performed as previously described [35]. After photosynthetic measurement, all measured leaves in the chamber were removed and carefully spread and photoed (Canon 5D Mark III, Canon Inc. Tokyo, Japan), and the surface area of each branch was calculated using CI-400 CIAS software (Computer Imaging Analysis Software, CID Co., Logan, UT, USA). The *P*_*n*_ and *T*_*r*_ were then converted to leaf-specific values. Diurnal *P*_*n*_ and *T*_*r*_ were measured from sunrise to sunset (7:00–22:00): measured at 1-h intervals during 7:00–12:00 and 16:00–22:00 to track rapid changes, and at 2-h intervals during 12:00–16:00.

A Model 1000 Pressure Chamber (PMS Instrument Company, Albany, OR, USA) was used to measure *Ψ*_*l*_ of the two species for every treatment at the same time as *P*_*n*_ and *T*_*r*_. Predawn leaf water potential (*Ψ*_*pd*_) was measured 30 min before sunrise, and midday leaf water potential (*Ψ*_*m*_) was measured at solar noon on the five irrigation interval treatments. Five replicate measurements were taken on small branches with enough leaves for each treatment. The instantaneous WUE (mmol CO_2_ mmol^−1^ H_2_O) was calculated as *P*_*n*_ (mmol CO_2_ m^−2^ s^−1^)/*T*_*r*_ (mmol m^−2^ s^−1^). The apparent hydraulic conductance of a plant is the change in *T*_*r*_ per change in *Ψ*_*l*_ driving the sap flow. The *Ψ*_*l*_ data against corresponding leaf-specific *T*_*r*_ to obtain the apparent hydraulic conductance for *H*. *ammodendron* and *C*. *mongolicunl* for the different irrigation intervals [21, 34, 41]. The slope of the linear relationship can be considered the leaf-specific apparent hydraulic conductance of the plant [42], while the X-axis intercept (*Ψ*_0_) is the water potential at the soil–root interface [21, 43]. The leaf-specific apparent hydraulic conductance of a plant represents the ability of the hydraulic system to transport water towards the leaves [34]. The data “before irrigation” and “after irrigation” were collected at five treatments on the days before and after the irrigation respectively.

### Measurement of leaf area and branch biomass

To quantify the leaf area of each branch, under each treatment, we photographed all foliage on each branch in the growing season every 2 weeks in 2014 (127–226 Julian d from the beginning of the year) when the treatments have been conducted for two years, five repetition for each treatment, the leaf surface area of each branch was calculated also as Xu and Li (2006) [35].

### Soil analysis and data statistics

The data of the soil water content was determined by oven-drying method [41]. The all variables are analyzed by standard statistical analysis. An ANOVA with Tukey’s HSD (honestly significant difference) (*P*<0.05) was used to determine the differences in the mean values of soil water content, root depth, plant biomass, and leaf area of per branch respectively. Regression analysis was used to determine the relationship between *Ψ*_*1*_ and *T*_*r*_. Figure preparation was done with Origin 8.0 (Origin LAB Corp., Northampton, MA, USA).

## Results

### Soil water content for different treatments

**Fig 2:**
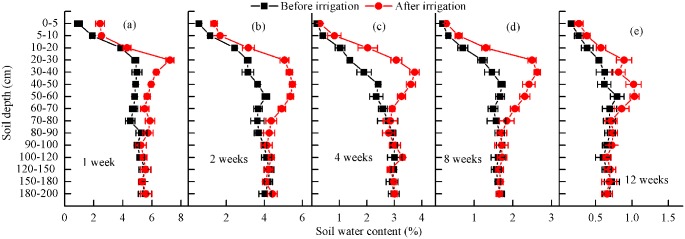
Vertical distribution of soil water content before and after the irrigation intervals of 1 (a), 2 (b), 4 (c), 8 (d) and 12 (e) weeks.

Irrigation frequency significantly influenced soil water content in the layer of 0–200 cm (*P*< 0.05; Fig 2). The results showed that soil water content of various layers significantly increased with increasing frequency of irrigation. After irrigation, it appeared significant soil water loss at the surface or upper layer since the strong evaporation in this area. Significant differences in the ground moisture content were also observed before and after irrigation for soil depth ≤ 80 cm, especially in 20–60 cm and remained stable for > 80 cm.

### Branch biomass change in the growing season

**Fig 3:**
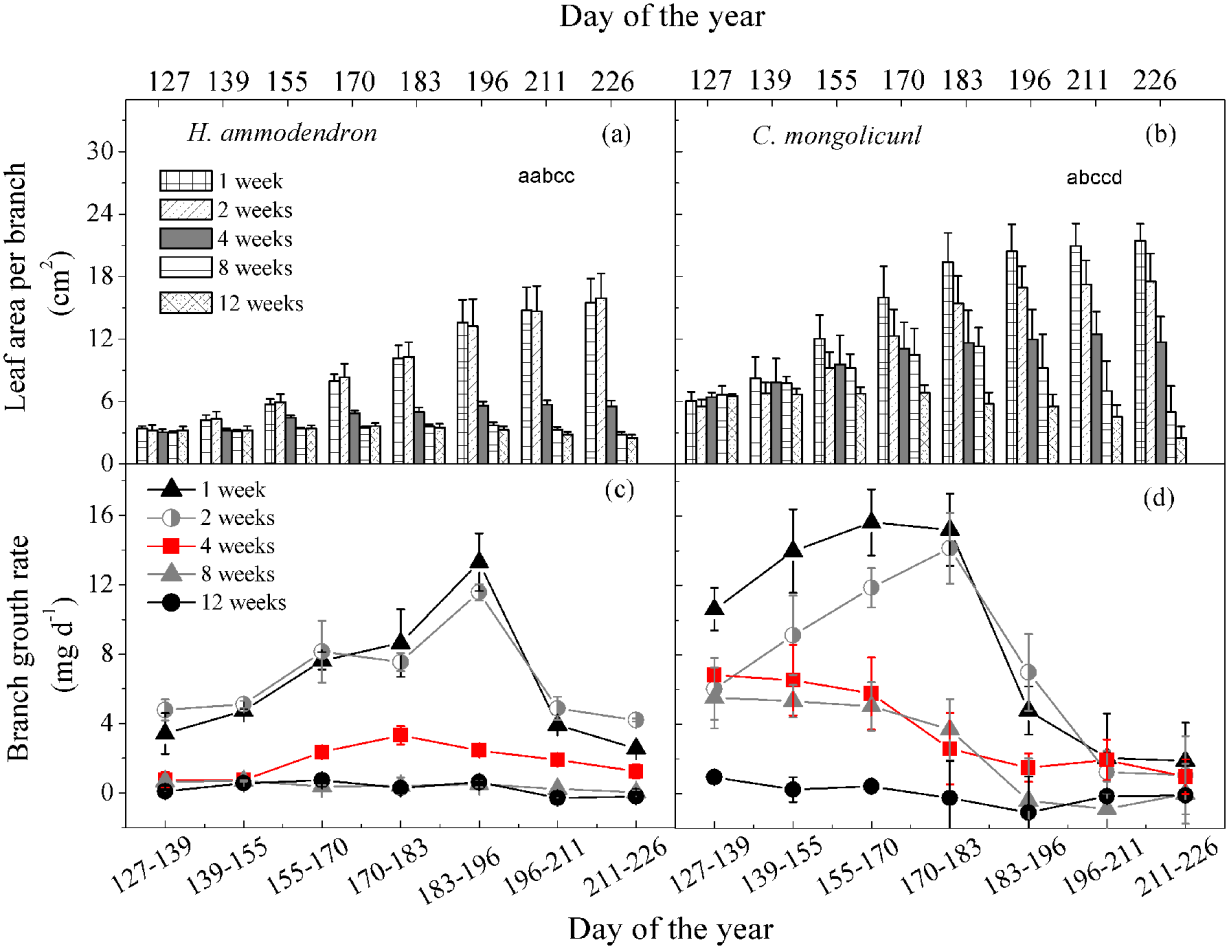
Seasonal changes in leaf area per branch and growth rate of branch biomass of *H*. *ammodendron* (a, c) and *C*. *mongolicunl* (b, d) for the five irrigation intervals. Error bars represent the standard deviation of the mean. Different lower-case letters indicate significant differences among treatments (*P* = 0.05).

Seasonal patterns of branch biomass and leaf area per branch for *H*. *ammodendron* and *C*. *mongolicunl* were significantly influenced by irrigation frequency. For both studied species, the leaf area per branch decreased with increasing irrigation interval (Table 2 and Fig 3). Moreover, the leaf area per branch for 8- and 12-week intervals in both species showed non-significant increases and even decreases during 127–226 d across the growing season (Table 2 and Fig 3). For *H*. *ammodendron*, the branch growth had an increasing trend during 127–196 d, and a decreasing trend during 197–226 d for 1- and 2-week treatments. Similar to 1- and 2-weeks, for 4-week treatment, the branch growth rate increased initially and then decreased. However, the growth rate was lower than 1- and 2-weeks. For *C*. *mongolicunl* the branch growth rate had a increasing trend during 127–183 d and a decreasing trend during 183–226 d for the 1- and 2-week treatment, for the 4-week treatment, although a decreasing trend for leaf growing rate presented in the growing season, the leaf area presented an increasing trend, while for the 8- and 12-week treatment, the branch growing rate and the leaf area also decreased during 183–226 d.

**Table 2:** Soil water content and biomass allocation of two species for 2-week irrigation interval at the TDBG and the TDSH where some *C*. *mongolicunl* have been dead.

	Soil water content (20 cm to the root depth) (%)	Biomass (g)				Branch	Assimilating leaf / Feeder root ratio	The ratios of root to stem
		Root depth (m)	Feeder root	Taproot	Leaf (Assimilating branch)			
*H*. *ammodendron*	4.58±035 a	1.7±0.43 a*	366±34b	1041±153 b	197±32b*	2583±298b*	0.538±0.047b *	0.506±0.026 b*
(TDBG)								
*H*. *ammodendron*	1.77±0.28A	3.6±0.65 A	342±37B	941±186B	169±29B	1898±257A	0.494±0.015 A	0.635±0.04B
(TDHS)								
*C*. *mongolicunl*	4.46±0.34 a *	0.85±0.15b *	994±52a*	2548±193 a*	891±103a*	3840±257a*	0.896±0.072a*	0.749±0.035 a*
(TDBG)								
*C*. *mongolicunl*	1.93±0.3A	2.2±0.52B	721±75A	1425±152 A	302±42A	1968±168A	0.419±0.025 B	0.945±0.054 A
(TDHS)								

Notes: the * represent significant differences of same species at two position, the different lowercases represent significant differences of two species at TDBG, and the different capital letters represent significant differences of two species at TDHS at *P* = 0.05.

### Aboveground and underground biomass allocation

Soil water content at the depth of 20 cm to the root depth showed that it was higher at the TDBG (Table 2), while in some parts of the TDHS, although the initial irrigation system was also set as 2 weeks interval, soil water content was only 1.7%-1.93% (Table 2), which was similar to our treatment of 8 weeks interval (Fig 2). The root depth and biomass for both species at TDBG and TDHS presented significant difference, and the primary root system for *H*. *ammodendron* extended to 1.7 m, while that for *C*. *mongolicunl* was no more than 1m at the TDBG. At some parts of the TDHS, they were about 3.6 m and 2.2 m respectively, which was deeper than they were at the TDBG. For *H*. *ammodendron* extended, there were no differences of the feeder root and taproot at TDBG and TDHS, but the assimilating branch and branch biomass at the TDBG were higher than in TDHS, the root to stem ratio (R/S) and the feeder root/ assimilating leaf ratio was enlarged from TDBG to TDHS. For *C*. *mongolicunl*, both underground and aboveground biomass were larger at TDBG than TDHS. Also, the root to stem ratio (R/S) and the feeder root/ assimilating leaf ratio was enlarged from TDBG to TDHS (Table 2).

### Physiological response to irrigation treatments

#### Leaf water potential and transpiration

**Fig 4:**
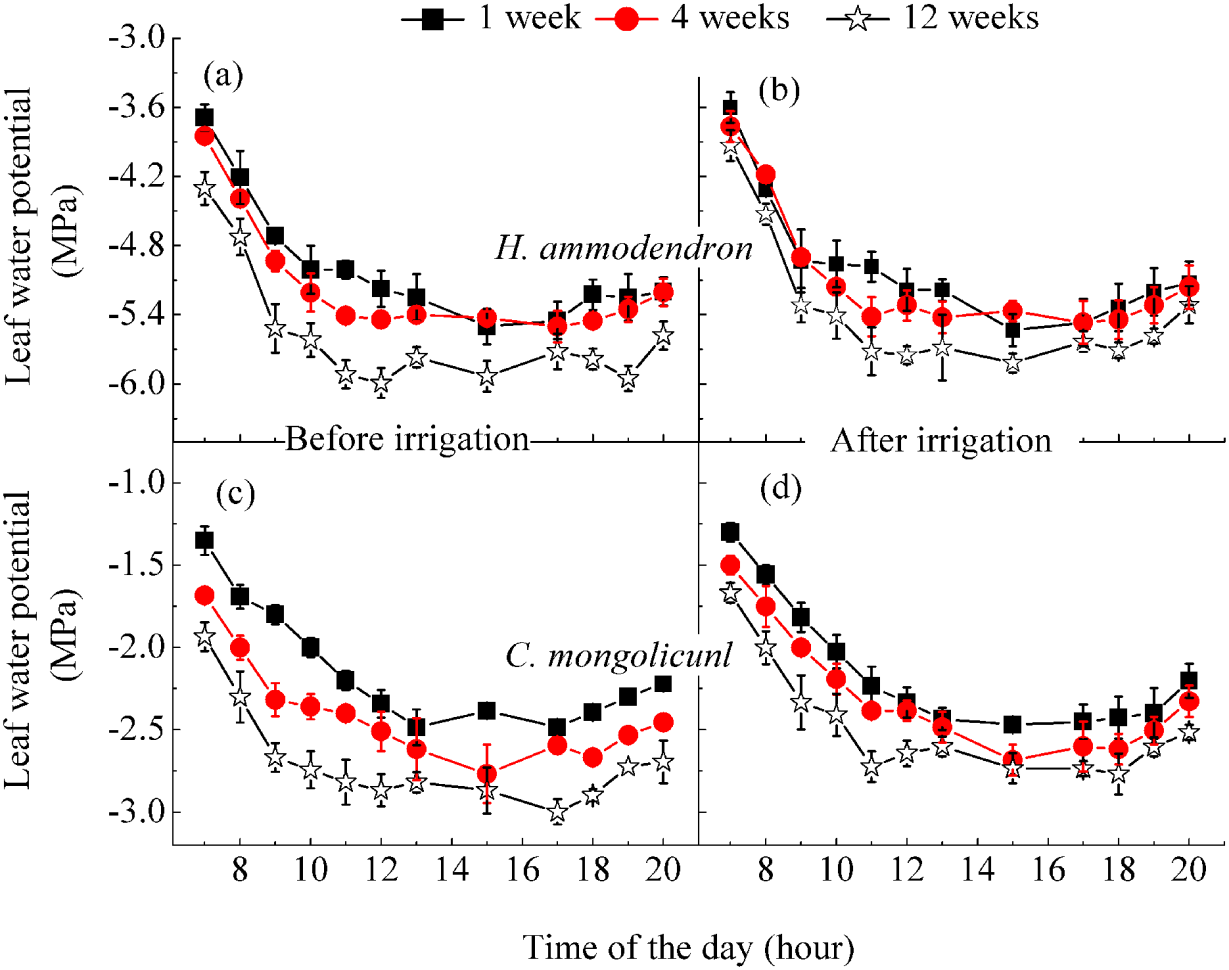
Diurnal pattern of leaf water potential of *H*. *ammodendron* (a, b) and *C*. *mongolicunl* (c, d) before and after irrigation for the five irrigation intervals. The data points for the 2-week interval fell between those of the 1 and 4 weeks; those of the 8-week interval fell between those of 4 and 12 weeks. Error bars represent the standard deviation of the mean.

The results showed that *Ψ*_*l*_ was higher for *C*. *mongolicunl* than *H*. *ammodendron* (Fig 4). For both species, both predawn leaf water potential (*Ψ*_*pd*_) and midday leaf water potential (*Ψ*_m_) had increasing trends with the lengthening of irrigation interval. Moreover, the difference between before and after irrigation increased with the prolongation of irrigation interval.

The diurnal course of *Ψ*_*1*_ for *H*. *ammodendron* (Fig 4a and 4b) remained at similar levels for the soil water contents of 1- and 4-week treatments both before and after irrigation, with only a slight difference of before and after for 12-week interval. Thus, the prolonged irrigation interval may have a strong influence on plant water availability. There were large significant differences in *Ψ*_*1*_ before irrigation of *C*. *mongolicunl* between 1, 4 and 12 weeks (Fig 4c), but after irrigation, the only large difference was between 1 and 12 weeks.

**Fig 5:**
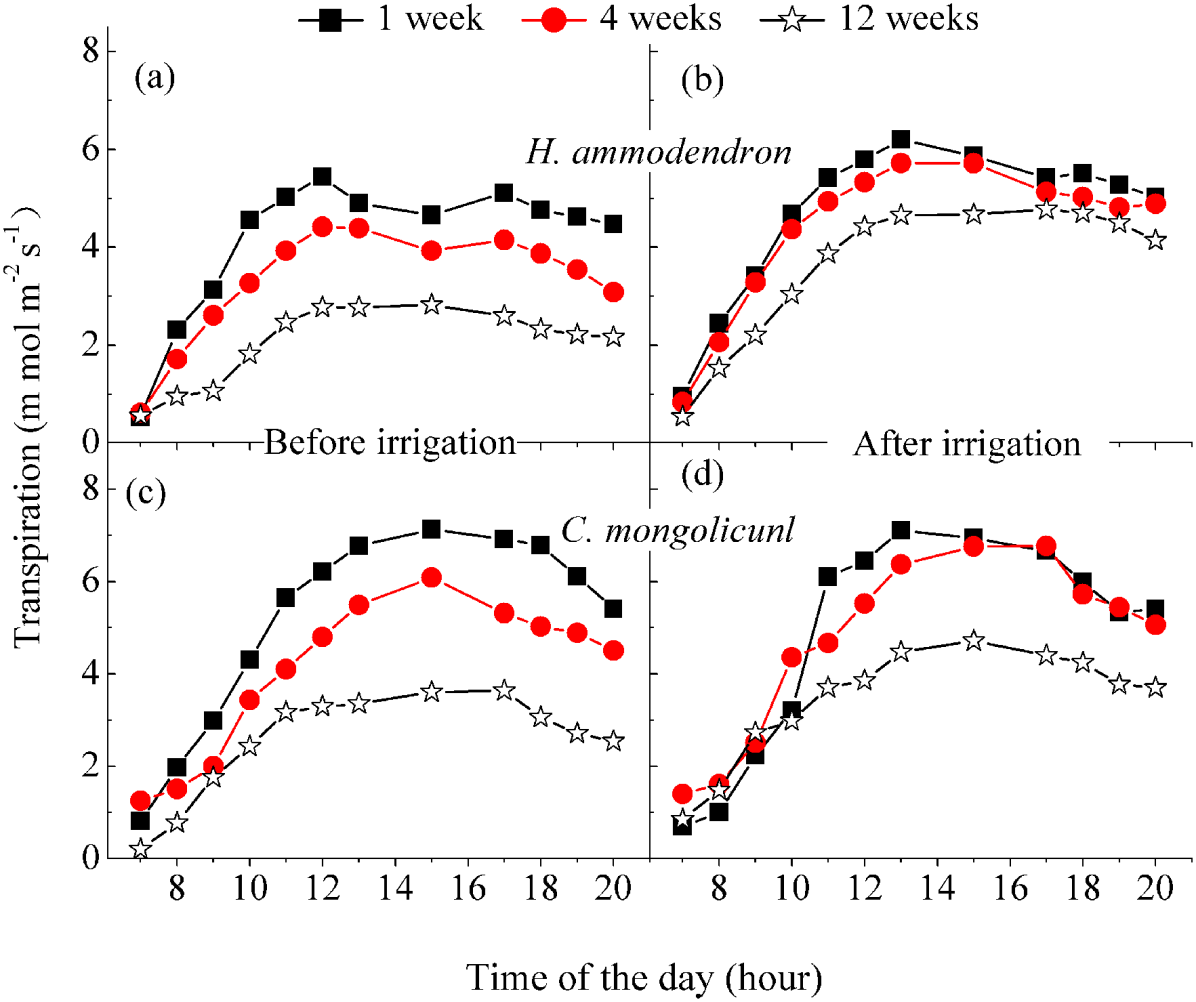
Diurnal pattern of transpiration for *H*. *ammodendron* (a, b) and *C*. *mongolicunl* (c, d) before and after irrigation for the five irrigation intervals. The data points for the 2-week interval fell between those of the 1 and 4 weeks; those of the 8-week interval fell between those of 4 and 12 weeks. Error bars represent the standard deviation of the mean.

The diurnal course of *T*_*r*_ for *H*. *ammodendron* and *C*. *mongolicunl* had an increasing trend during 7:00–12:00 am, and it presented urgently increasing trend and then a decreasing trend until sunset during 12:00–18:00 (Fig 5b and 5d). Additionally, *T*_*r*_ remained at similar levels for the soil water contents after 1- and 4-week irrigation, with only a small difference after the 12-week irrigation (Table 3). There were significant differences between 1-, 4- and 12-week treatments for before irrigation in both plants (Fig 5a and 5c and Table 3), indicating that under drought stress (i.e. before irrigation) there were significant effects on transpiration for both plants. Furthermore, there was no significant difference in *T*_*r*_ for both plants between before and after 1-week irrigation, but there were significant differences for 4 and 12 weeks (Fig 5 and Table 3).

**Table 3:** Comparison of mean physiological and growth parameters of *Haloxylon ammodendron* and *Calligonum mongolicunl* before and after irrigation with intervals of 1, 4 and 12 weeks.

Species	Parameters	1 week		4 weeks		12 weeks	
		Before irrigation	After irrigation	Before irrigation	After irrigation	Before irrigation	After irrigation
*H*. *ammodendron*	*Ψ*pd (MPa)	-3.69C	-3.60b	-3.85B	-3.77ab	-4.30A	-3.93a*
	*Ψ*m (MPa)	-5.25B	-5.18b	-5.44B	-5.42ab	-5.77A	-5.68a*
	*Pn* (μ mol CO_2_ m^-2^s^-1^)	10.24C	10.76c	8.92B	9.93b	6.29A	8.17a*
	*Tr* (m mol m^-2^s^-1^)	4.03C	4.52b	3.17B	4.25b*	2.00A	3.49a*
	WUE (μ mol CO_2_ mmol H_2_O)	2.39A	2.248a	2.58A	2.27a	3.02B	2.44a*
	Leaf area (cm^2^)	15.5c		5.55b		2.52a	
	Branch growth rate (mg d^-1^)	6.31c		1.81b		0.25a	
*C*. *mongolicunl*	*Ψ*pd (MPa)	-1.35C	-1.3b	-1.68B	-1.5ab	-1.93A	-1.67a*
	*Ψ*m (MPa)	-2.48C	-2.43b	-2.62B	-2.48ab	-2.82A	-2.60a*
	*Pn* (μ mol CO_2_ m^-2^s^-1^)	13.69C	15.22c*	10.9B	12.74b*	5.05A	8.55a*
	*Tr* (m mol m^-2^s^-1^)	4.90C	4.85b	3.91B	4.52b*	2.49A	3.33a*
	WUE (μ mol CO_2_ mmol H_2_O)	2.72C	3.23c*	2.68B	2.89b*	2.02A	2.45a*
	Leaf area (cm^2^)	21.43c		11.71b		2.49a	
	Branch growth rate (mg d^-1^)	9.20c		3.7b		0.00a	

Notes: the values of 2 weeks interval was between the 1 week and 4 weeks and near the 1 week, 8 weeks interval was between 4 weeks and 12 weeks and near the 12 weeks.

* represents a significant difference (*P* = 0.05) between before and after irrigation at the same interval; a capital letter indicates a significant difference (*P* = 0.05) before irrigation at the different intervals; and a lower-case letter indicates a significant difference (*P* = 0.05) after irrigation at the different intervals.

### Photosynthesis rates and water-use efficiencies

**Fig 6:**
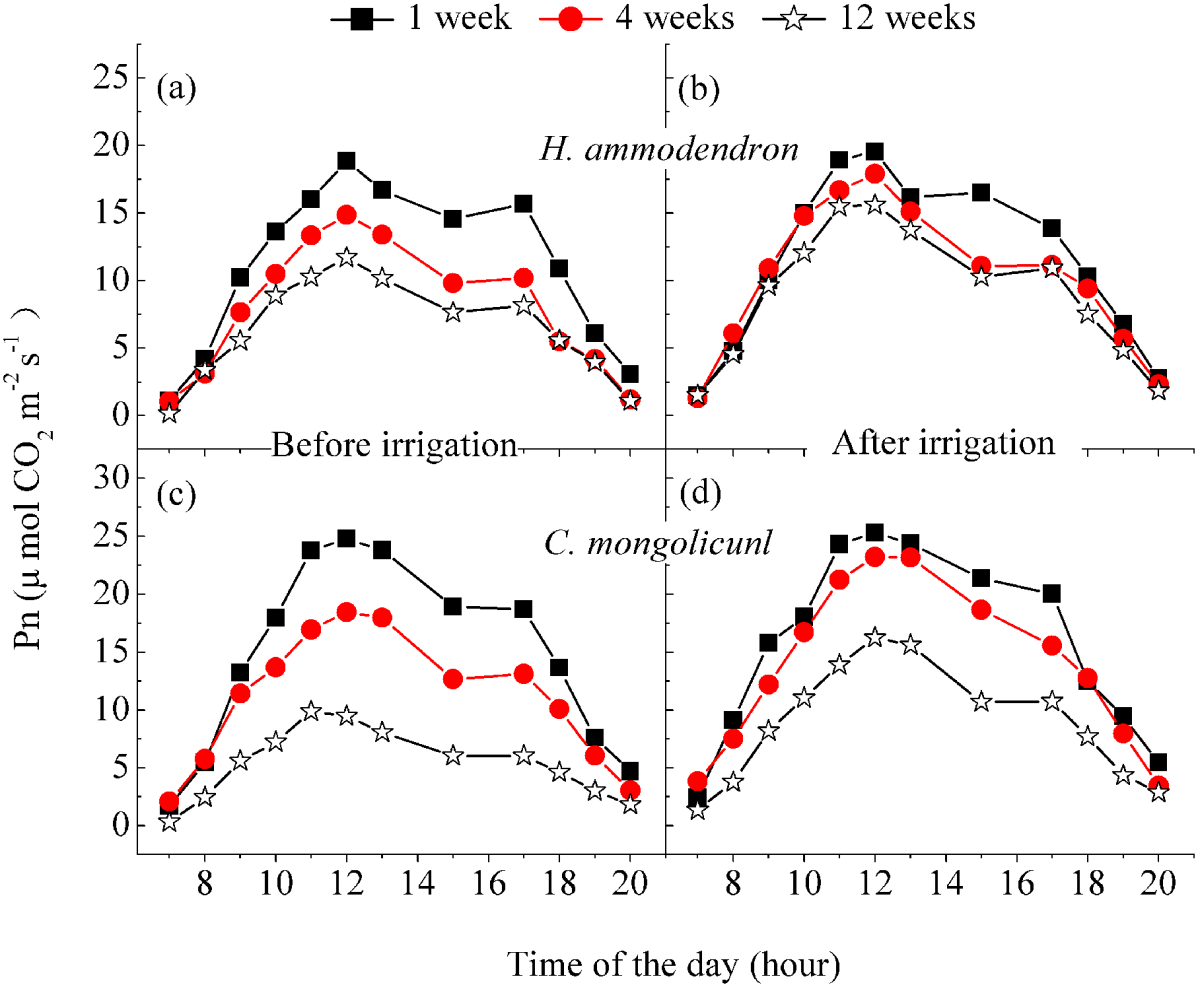
Diurnal photosynthesis patterns of *Haloxylon ammodendron* (a, b) and *Calligonum mongolicunl* (c, d) before and after irrigation for the five irrigation intervals. The data points for the 2-week interval fell between those of the 1 and 4 weeks; those of the 8-week interval fell between those of 4 and 12 weeks. Error bars represent the standard deviation of the mean. Different lower-case letters indicate significant differences among treatments (*P* = 0.05).

Diurnal *P*_*n*_ of *H*. *ammodendron* and *C*. *mongolicunl* showed an increasing trend during 7:00–11:00, a midday depression during 12:00–16:00 and then decreased until sunset (Fig 6 and Table 3). The *P*_*n*_ of *C*. *mongolicunl* differed significantly before and after irrigation (*P*< 0.05) (Fig 6c and 6d and Table 3). However, for *H*. *ammodendron P*_*n*_, it did not significantly differ before and after irrigation except for the 12-week interval. This result indicated that *C*. *mongolicunl* was vulnerable to soil water deficit in this area, similarly to the other physiological characteristics, *Ψ*_*l*_ and *T*_*r*_. There was little difference in *P*_*n*_ for *H*. *ammodendron* between1-, 2- and 4-week treatments. We, therefore, conclude that *P*_*n*_ of *H*. *ammodendron*, at the leaf scale, was not inhibited by 1-, 2- and 4-week irrigation; however, for the 12-week interval, *P*_*n*_ was inhibited significantly for both shrubs. The water use efficiency (WUE) of *H*. *ammodendron* remained steady except for before and after 12-week treatment, during which it experienced drought stress before the irrigation–WUE was much higher than for the other treatments. For *C*. *mongolicunl*, WUE was always higher before irrigation for all treatments (Table 3), showing that *C*. *mongolicunl* was more sensitive to water.

### Hydraulic conductance

**Fig 7:**
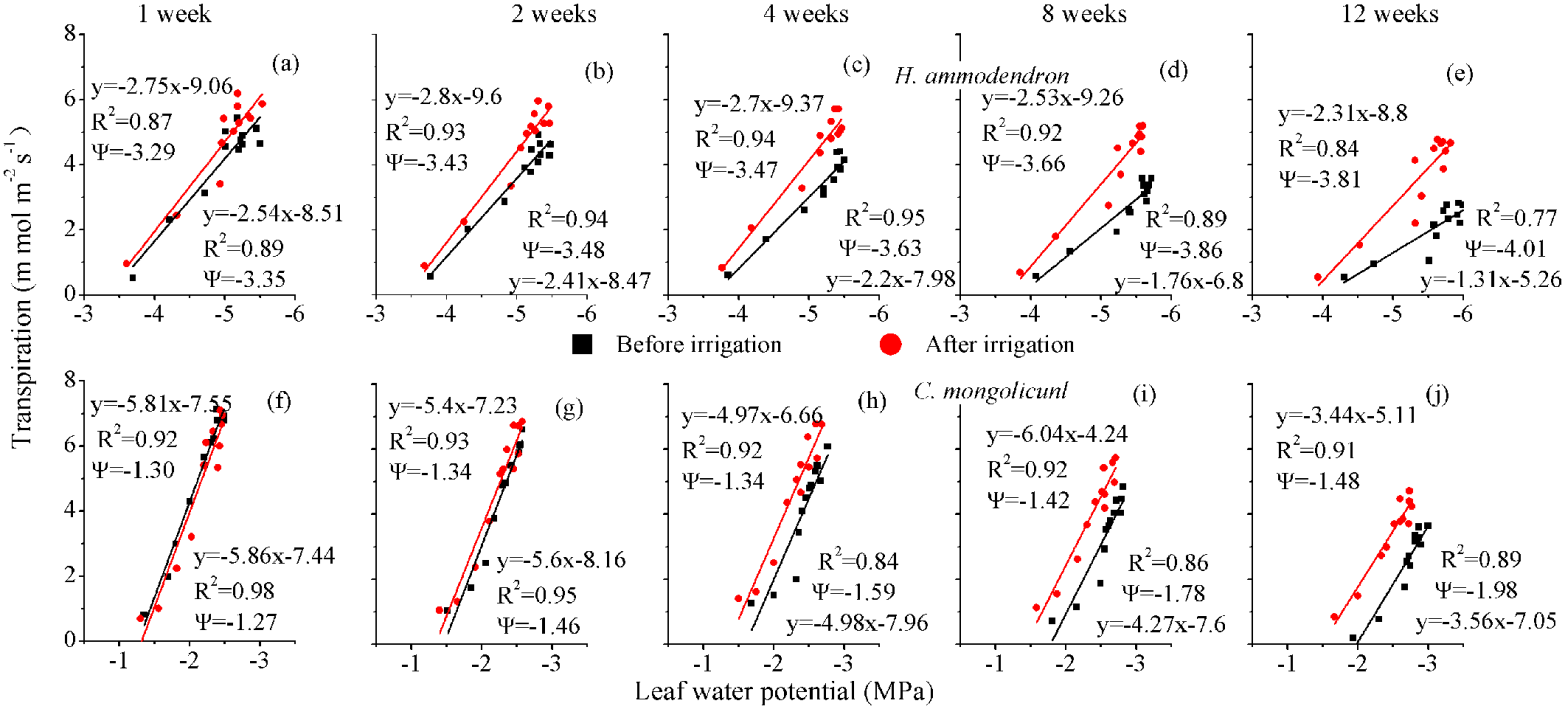
Leaf-specific apparent hydraulic conductance of *Haloxylon ammodendron* (a–e) and *Calligonum mongolicunl* (f–j) before and after irrigation with intervals of 1, 2, 4, 8 and 12 weeks. The relationship between leaf water potential and transpiration rate was best fitted by the linear function Y = A + BX, in which A represents *Ψ*_0_ which is the water potential at the soil–-root interface and B represents leaf-specific apparent hydraulic conductance before and after irrigation.

We plotted the *Ψ*_*l*_ data (Fig 4) against corresponding leaf-specific *T*_*r*_ to obtain the apparent hydraulic conductance for *H*. *ammodendron* (Fig 7a–7e) and *C*. *mongolicunl* (Fig 7f–7j) for the different irrigation intervals. The value was 2.7–2.8 mmol m^−2^s^−1^ MPa^−1^ for *H*. *ammodendron* after irrigation of 1-, 2- and 4 week intervals, and 2.5 and 2.3 mmol m^−2^s^−1^ MPa^−1^ after irrigation of 8- and 12-week intervals, respectively (Fig 6). Our results reported that before irrigation, the value was lower, in the range of 1.31–2.54 mmol m^−2^s^−1^MPa^−1^. For *C*. *mongolicunl*, the value was 5.8 mmol m^−2^s^−1^ MPa^−1^ for both before and after irrigation of 1-week interval (Fig 7f), with a decreasing trend for prolonging of irrigation interval from 5.81 to 3.44 mmol m^−2^s^−1^ MPa^−1^ after irrigation, and from 5.86 to 3.56 mmol m^−2^s^−1^ MPa^−1^ before irrigation. Moreover, for both species, the difference between the values increased with the prolonging of irrigation interval.

Besides, for *H*. *ammodendron* (Fig 7a–7e), the slope differed between before and after irrigation in each treatment, while *Ψ*_0_ remained constant. However, for *C*. *mongolicunl* (Fig 7f–7j), the slope remained consistent before and after irrigation in each treatment, while *Ψ*_0_ differed.

## Discussion

### Morphological adjustment

Morphological adjustments in desert shrubs can maintain the balance between water supply and demand in a plant through the plant root and shoot distribution [25, 34, 42]. Our results showed that the adjustment was linked with the ontogenetic structural change, hydraulic architecture adjustment and individual carbon allocation at the roots and shoots. In good water conditions (1- and 2-week irrigation intervals), carbon assimilation was significantly higher than for other irrigation treatments (Fig 3a and 3b), which has also been documented under the variation in summer precipitation in the Gurbantonggut Desert [26, 35]. The photosynthetic capacity changed significantly at the leaf scale for drought status and changed little for good water conditions (Fig 6). The carbon assimilation ability was greater for *C*. *mongolicunl* than *H*. *ammodendron* in good water condition, and more photosynthetic products were allocated to roots for both species, especially for *C*. *mongolicunl* (Fig 2 and Table 2). The root/shoot ratio of *C*. *mongolicunl* was higher than of *H*. *ammodendron*, while the assimilating leaf/feeder root ratio was opposite (Table 2); under water shortage conditions (at TDHS or 8- and 12-week irrigation intervals), there was much defoliation in response to drought. With moderate irrigation interval (4 weeks), the assimilative organs grew gently and no significant defoliation was observed through the growth season.

In particular, shoot size may shrink by partial defoliation so that the root system can support it, which represents another adaptation in response to drought [25]. In this case, the preferential belowground allocation of resources may limit the shoot (canopy) investment, accompanying the defoliation of assimilative organs. This morphological adjustment maintained the balance between water supply to the root system and water demand of the shoot system, which ensured a consistent photosynthetic capacity of surviving leaves [22, 25]. However, the carbon assimilation of the individual plant was significantly reduced by the water stress of 12-week interval for *C*. *mongolicunl*. Defoliation and the higher quota for the root system are two sides of the morphological trade-off that ensures normal photosynthesis and subsistence. If the balance between water supply to the root system and water demand of the shoot system is broken, the limited assimilative organs cannot supply sufficient nutrition to roots, and the plants may starve [25, 43, 44]. This may be the cause for the death of *C*. *mongolicunl* in some parts of the TDHS (Fig 1a).

For *H*. *ammodendron*, although the R/S ratio was smaller than when growing in the native desert, while the assimilating leaf/feeder root ratio was larger than the eigenvalue ratio of 0.4 when it is growing in moderate water condition in the original sites [29]. This result documented that the two-week interval lead to good water conditions for *H*. *ammodendron* than it grows in the native desert [29]. Furthermore, compared with *C*. *mongolicunl*, the *H*. *ammodendron* root system was deeper, and thus it could access deeper water during drought stress. Most importantly, the larger assimilating leaf/feeder root ratio indicated that the increase of the leaf area (assimilating branches) is another way to gain more carbon assimilation products in stress environments [45]. Such morphological adjustment contributed significantly to the strong drought tolerance of *H*. *ammodendron* and ensured its natural photosynthesis and survival at the physiological scale.

### Physiological response

The *Ψ*_*l*_, *P*_*n*_, *T*_*r*_ and WUE of both species depended on irrigation, especially for the 12-week interval (Table 3) (*P* < 0.05). The lower WUE before the 1- and 4-week irrigations suggested a lack of tight stomata control under favorable water conditions (Fig 4b) [22]. Before irrigation or with the prolonged irrigation interval, *T*_*r*_ decreased at times of depleted soil water (Table 3), indicating efficient stomatal control under drought stress [29]. The significantly enhanced *T*_*r*_ when drought stress was relieved, reported the capacity of stomatal control under drought stress–this is characteristic of actively drought-tolerant plants [26].

With decreasing soil water, *Ψ*_pd_ and *Ψ*_m_ had a decreasing trend (Fig 4), while WUE had an increasing trend (Table 3), showing that high sensitivity to water availability ensured an increase in WUE under drought stress (Table 3). A close correlation (*R*^*2*^> 0.9) between *T*_*r*_ and *Ψ*_*1*_ when *Ψ*_*1*_> −5.5 Mpa for *H*. *ammodendron* and *Ψ*_*l*_ > −2.7 MPa for *C*. *mongolicunl* demonstrates the immediate impact of *Ψ*_*1*_ on stomatal conductance with good soil water content. The correlation declined significantly (*R*^*2*^ = 0.77) (Fig 7e) when *Ψ*_*l*_ remained relatively low, indicating that for drought stress conditions, stomata conductance was directly affected by factors other than *Ψ*_*l*_. This result was inconsistent with the results of Xu and Li (2006) [35]. However, if the root system cannot get the water soon, the available water will be depleted, and the *Ψ*_*l*_ of surviving leaves will decrease beyond the lower limit, and in this case, photosynthesis and the survival of plants will be threatened [45, 46], which is consistent with the morphological adjustment for *C*. *mongolicunl* in our result.

The consistent values of leaf-specific apparent hydraulic conductance indicate that the transporting capacity of the *C*. *mongolicunl* hydraulic system did not change in response to irrigation (Fig 7f–7j). The changed values of leaf-specific apparent hydraulic conductance indicated that the transporting capacity of *H*. *ammodendron* changed significantly in response to irrigation, which was inconsistent with Xu and Li’s results [35]. *Ψ*_*0*_ was different for *C*. *mongolicunl*, which changed from −1.27 to −1.98 MPa under different treatments; with prolonged irrigation interval, the difference increased, and it also differed significantly before and after irrigation of 4-, 8- and 12-week intervals (Fig 7). Consistent slopes at before and after irrigation in every treatment, indicating that the soil water status sensed by the root system improved significantly. These observations suggest that *C*. *mongolicunl* was able to maintain a stable water transporting capability from root to foliage, regardless of soil water status in the root zone. While for *H*. *ammodendron*, *Ψ*_*0*_ was consistent before and after irrigation in the same treatment, but slopes changed–indicating that the leaf-specific apparent hydraulic conductance of *H*. *ammodendron* changed significantly, while water potential at the soil–root interface remained constant for different water conditions. These results are not consistent with those of Xu et al. (2007) [29], which may be the unique adjustment mechanism for *H*. *ammodendron* through changing the leaf-specific apparent hydraulic conductance when it was introduced to the TDHS and TDBG, and demonstrated that *H*. *ammodendron* possessed a constant leaf transporting capacity in good water conditions, and changed significantly under drought stress.

The diurnal course of *Ψ*_*l*_ showed an opposite situation to *Pn* and *T*_*r*_—the high temperature at midday restricted physiological activity and induced stomatal closure [18, 35, 46]. Additionally, *Pn* and *T*_*r*_ presented similar situations between the different irrigation intervals; the physiological activity and transpiration for both plants were significantly higher under good water conditions (before and after 1- and 4-week irrigation intervals). These results demonstrated the efficiency of physiological adjustment for the two species in this extreme environment–as the higher *T*_*r*_ after irrigation (Fig 5) also produced a higher photosynthetic rate, likely through effective osmotic adjustment [29].

### Relationship between physiological responses and morphological adjustment

The psammophyte, *C*. *mongolicunl*, had a shallow but horizontal and larger root system and was more susceptible to water supply than *H*. *ammodendron* [31]. In contrast, both plants are xerophytes and had strong responses to increased water supply and efficiencies in morphological adjustment, physiological characteristics response that will benefit their growth, survival, and competition in extreme arid habitats [22, 46]. However, when the balance between water supply to the root system and water demand of the shoot system is broken, the limited assimilative organs restrict their photosynthesis ability and cannot provide sufficient nutrition to roots, and the plants may starve (Fig 1a) [18, 25].

Previous studies have documented that *H*. *ammodendron* maintained a variable water use strategy in its natural habitat [4, 26, 29, 33]. It mainly uses shallow soil water when the upper soil water is abundant in early spring due to melting snows, while in summer when the upper soil water is depleted, *H*. *ammodendron* mainly uses groundwater [4, 33]. Our results demontrate that *H*. *ammodendron* had a relatively deep root system (Table 3), and roots might reach the ground water when water scarcity is serious [4, 41,47]. The significant *Pn*, *T*_*r*_ and *Ψ*_*l*_ responses of *C*. *mongolicunl* to irrigation intervals of 4, 8 and 12 weeks (Fig 4) indicate that the root system sensed water conditions following irrigation, and the consistency of leaf-specific conductance with or without irrigation (Fig 7f–7j) suggests that *C*. *mongolicunl* maintained a balance between water supply of the soil–root system and water demand of the atmosphere–shoot system [36]. One possible mechanism behind this phenomenon is that the root development was not efficiently adjusted as the root zone progressively dried [38]. Another possibility is that shoot size was reduced via partial defoliation in response to soil dehydration.

## Conclusion

The comparison among treatments in this study indicates that variations in irrigation interval significantly affected the individual-scale carbon gain and biomass allocation pattern of both plants. The variation in water will change the existing water-use strategy of the root system and, consequently, the architecture of the whole plant. *C*. *mongolicunl is* more sensitive to drought because of a shallow root system and preferential belowground allocation of resources, which documented our first hypothesis that morphological adjustment is the main cause for the death of *C*. *mongolicunl*. At the same time, *H*. *ammodendron* possesses a variable leaf-specific conductance, while that of *C*. *mongolicunl* is stable. The combination of the above trends indicates that a moderate irrigation interval of 4 weeks was suitable for both plants since it not only saved water but also met the water demands of the plants.
